# Nanostructured Bi_2_Te_3_ Prepared by a Straightforward Arc-Melting Method

**DOI:** 10.1186/s11671-016-1345-5

**Published:** 2016-03-15

**Authors:** M. Gharsallah, F. Serrano-Sánchez, J. Bermúdez, N. M. Nemes, J. L. Martínez, F. Elhalouani, J. A. Alonso

**Affiliations:** Instituto de Ciencia de Materiales de Madrid, C.S.I.C., Cantoblanco, E-28049 Madrid Spain; National School of Engineers, Sfax University, Sfax, B. P. W 3038 Tunisia

**Keywords:** Thermoelectrics, Nanostructuration, Lattice thermal conductivity, Thermopower, Neutron powder diffraction, ZT figure of merit

## Abstract

Thermoelectric materials constitute an alternative source of sustainable energy, harvested from waste heat. Bi_2_Te_3_ is the most utilized thermoelectric alloy. We show that it can be readily prepared in nanostructured form by arc-melting synthesis, yielding mechanically robust pellets of highly oriented polycrystals. This material has been characterized by neutron powder diffraction (NPD), scanning electron microscopy (SEM), and electronic and thermal transport measurements. A microscopic analysis from NPD data demonstrates a near-perfect stoichiometry of Bi_2_Te_3_ and a fair amount of anharmonicity of the chemical bonds. The as-grown material presents a metallic behavior, showing a record-low resistivity at 320 K of 2 μΩ m, which is advantageous for its performance as a thermoelectric material. SEM analysis shows a stacking of nanosized sheets, each of them presumably single-crystalline, with large surfaces perpendicular to the *c* crystallographic axis. This nanostructuration notably affects the thermoelectric properties, involving many surface boundaries that are responsible for large phonon scattering factors, yielding a thermal conductivity as low as 1.2 W m^−1^ K^−1^ around room temperature.

## Background

Thermoelectric materials can convert temperature gradients, prominently those generated by waste heat, into useful electrical power [1, 2]. As thermoelectric generators are scalable and present advantages such as no loose parts and reliability, they could play an important role in the sustainable development [3]. Among thermoelectric materials, Bi_2_Te_3_-related alloys have shown the best thermoelectric performance for n-type systems at room temperature, thus they are also the most commercially used [4–9]. Thermoelectric properties are assessed in terms of the figure of merit, ZT, defined as ZT = *S*^2^*σT*/*κ*, where *S* accounts for the Seebeck coefficient, *σ* is the electrical conductivity, *κ* is the thermal conductivity, and *T* is the absolute average temperature. Optimization of these physical properties is a challenging task as they depend on strongly correlated physical parameters.

Recent work on thermoelectric materials revealed that nanostructured bulk samples could provide highly improved energy conversion [10, 11]. Latest advances maximizing the figure of merit present bulk nanostructured materials displaying enhanced efficiency mainly through reduction of *κ* by phonon localization, by increased phonon scattering in grain boundaries, and the improvement of *σ* and *S* by changing the density of states [12].

In bulk pristine Bi_2_Te_3_, the thermoelectric performance depends on the microstructural arrangement of grains, element distribution, and composition [3, 9]. These parameters are essentially controlled by the synthesis and preparation methods of the samples. Many different techniques have been employed in the production of nanostructured Bi_2_Te_3_ alloys, like laser ablation, mechanical alloying and hot pressing, vapor deposition, and ligand-assisted wet chemical methods [13–15]. Mechanical and physical treatments commonly involve high-energy consumption and several steps; on the other hand, chemical methods present lower ZT values due to inappropriate carrier concentration and low intergranular connectivity [16, 17].

In this paper, we describe a straightforward preparation procedure of compact pellets of pure Bi_2_Te_3_, by arc melting from mixtures of the starting elements, in truly short reaction times, leading to highly oriented polycrystalline samples for which we found low thermal conductivities, probably linked to the nanostructured nature of the polycrystalline domains. We report on a complete characterization including transport (Seebeck, electrical and thermal conductivity, and Hall effect) and crystal structure from neutron powder diffraction (NPD) data to assess the microscopic nature of the raw material.

## Methods

Bi_2_Te_3_ was prepared in an Edmund Buhler mini-arc furnace. A mixture of stoichiometric amounts of Bi (99.999 %, Cerac) and Te (99.999 %, Alfa Aesar) was molten under Ar atmosphere in a water-cooled Cu crucible, leading to intermetallic ingots, which were ground to powder for structural characterization, or cut with a diamond saw in bar-shape for transport measurements.

The reaction products were characterized by X-ray diffraction (XRD) with Cu K_α_ radiation using a Bruker-AXS D8 diffractometer (40 kV, 30 mA), controlled by DIFFACT^PLUS^ software, in Bragg-Brentano reflection geometry with Cu K_α_ radiation (*λ* = 1.5418 Å). A room-temperature NPD pattern was collected at the HRPT diffractometer of the SINQ spallation source (Switzerland), with a wavelength *λ* = 1.494 Å. The sample was packed in a cylindrical vanadium holder (dia. 8 mm), and the counting time was 2 h in the high-intensity mode; the sample holder was rotating during the acquisition time. The refinement of the structure was performed by the Rietveld method and the FULLPROF refinement program [18]. A Thompson-Cox-Hastings pseudo-Voigt function was chosen to generate the line shape of the diffraction peaks. The coherent scattering lengths used for Bi and Te were, respectively, 8.532 and 5.80 fm. A preferred orientation correction was applied, considering platelets perpendicular to the [0 0 1] direction. No regions were excluded in the refinement. The following parameters were refined in the final runs: scale factor, background coefficients, zero-point error, pseudo-Voigt corrected for asymmetry parameters, occupancy of Bi and Te, positional coordinates and anisotropic displacements. High-resolution FE-SEM images were recorded in a FEI-Nova microscope.

The Seebeck coefficient and electrical resistivity were determined with a home-made device. Measurements were carried out under high vacuum (10^−5^ Torr), in the temperature range of 300–700 K. Resistivity was determined by a four-wire method while Seebeck coefficient was measured applying a constant temperature gradient across the sample of 20 K. Disks of 10 mm diameter with perfectly parallel faces were obtained from the as-grown ingots treated under an isostatic pressure of 10^3^ psi. The resistivity and the Seebeck coefficient were measured in these pressed pellets, along the axial pressing direction, whereas the thermal conductivity was measured in pellets cut from the as-grown ingots, perpendicular to the pressing direction.

Thermal conductivity was determined in a thermal transport setup within a Physical Properties Measurement System (PPMS) by Quantum Design. The measurements were carried out in the residual vacuum of He atmosphere, under a pressure of 10^−5^ Torr, in the temperature range of 2 to 380 K. The typical size of a Bi_2_Te_3_ pellet was 10 × 3 × 2 mm^3^ with four Cu wires attached with Ag paste. A constant temperature gradient of 3 % was applied across the sample during the whole measurement process. The thermal conductivity was measured perpendicular to the pressing direction and parallel to the pellet face that contacts with the Cu crucible during the preparation.

The Hall effect was determined using a four-wire technique in the van der Pauw geometry from a pellet of 8 × 3 × 3 mm^3^ dimensions. The sample was mounted on a horizontal rotator and the Hall resistance determined from the sinusoidal variation with the angle between a large applied magnetic field of 8.5 T and the plane of the sample [19].

## Results and Discussion

**Fig. 1:**
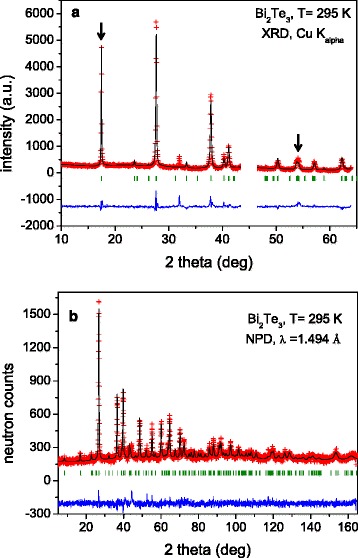
**a** XRD pattern for as-grown Bi_2_Te_3_, Rietveld-refined in the space group *R-3m*. A strong preferred orientation is observed, enhancing the [0 0 l] reflections, marked with an *arrow*. **b** Observed (*crosses*), calculated (*full line*), and difference (at the *bottom*) NPD profiles for Bi_2_Te_3_ at RT

The sample was obtained as a compact pellet with metallic luster which was handled in a glove box under N_2_ atmosphere. A Bi_2_Te_3_-type structure defined in the space group *R-3m* was identified *via* XRD. Diffraction patterns (Fig. 1a) show a strong preferred orientation enhancing the [0 0 l] reflections, already indicating a strong texture for the as-grown sample. A preferred orientation function was introduced to improve the match between simulated and observed profiles. The density of the samples, from the geometric parameters, is 7.1 g cm^−3^ for the as-grown ingots directly obtained from the arc furnace, while the density of the pressed disks is 8.0 g cm^−3^. The crystallographic density, *ρ*_x_, is 7.84 g cm^−3^ [20].

A neutron powder diffraction (NPD) study was essential to investigate the structural details of Bi_2_Te_3_. The bulk analysis provided by neutrons, and the rotation of the sample holder during the experiments hugely minimized the preferred orientation effect, accomplished by grinding the crystals to powder and packing them in vanadium cylinders. Additionally, neutrons permit to access a much wider range of the reciprocal space, and furthermore, the lack of a form factor allows determining precisely the anisotropic displacement factors. The crystal structure refinement from the NPD data at RT was carried out in the Bi_2_Te_3_-type model [21] in the hexagonal setting of the rhombohedral R-3m space group (no. 12), *Z* = 3, with Bi located at 6*c* (0 0 z) positions and the two types of tellurium, Te1 at 3*a* (0 0 0) positions and Te2 at 6*c*. The occupancy factors of Bi vs Te (with respect to Te at 6*c* position fixed to unity) show a small deviation from the initial stoichiometry. There was an excellent agreement between observed and calculated profiles, as shown in Fig. 1b; a minor preferred orientation correction was effective in improving the refinement for all the reflection in the whole angular range. Tables 1 and 2 include the atomic parameters and displacements factors, as well as the discrepancy factors after the refinement.

**Table 1:** Fractional atomic coordinates and isotropic or equivalent isotropic displacement parameters (Å2) for Bi_2_Te_3_ refined in the R-3m space group (hexagonal setting) from NPD data collected at RT. Unit cell parameters: *a* = 4.385915 (6) Å, *c* = 30.495497 (1) Å, *V* = 508.03 (5) Å^3^, *Z* = 3. The discrepancy factors after the refinement are also included

	*x*	*y*	*z*	*U*_iso_/*U*_eq_
Bi	0.00000	0.00000	0.4003 (2)	0.0147 (16)
Te1	0.00000	0.00000	0.00000	0.016 (4)
Te2	0.00000	0.00000	0.7900 (2)	0.031 (5)

Discrepancy factors: *R*_p_ = 5.56 %, *R*_wp_ = 7.03 %, *R*_exp_ = 6.35 %, *R*_Bragg_ = 9.45 %

**Table 2:** Anisotropic displacement parameters (Å2) for Bi_2_Te_3_ after the refinement described in Table 1

	*U* ^11^	*U* ^22^	*U* ^33^	*U* ^12^	*U* ^13^	*U* ^23^
Bi	0.0105 (8)	0.0105 (8)	0.023 (3)	−0.0052 (8)	0.00000	0.00000
Te1	0.016 (2)	0.016 (2)	0.017 (7)	−0.008 (2)	0.00000	0.00000
Te2	0.031 (3)	0.031 (3)	0.030 (7)	−0.016 (3)	0.00000	0.00000

**Fig. 2:**
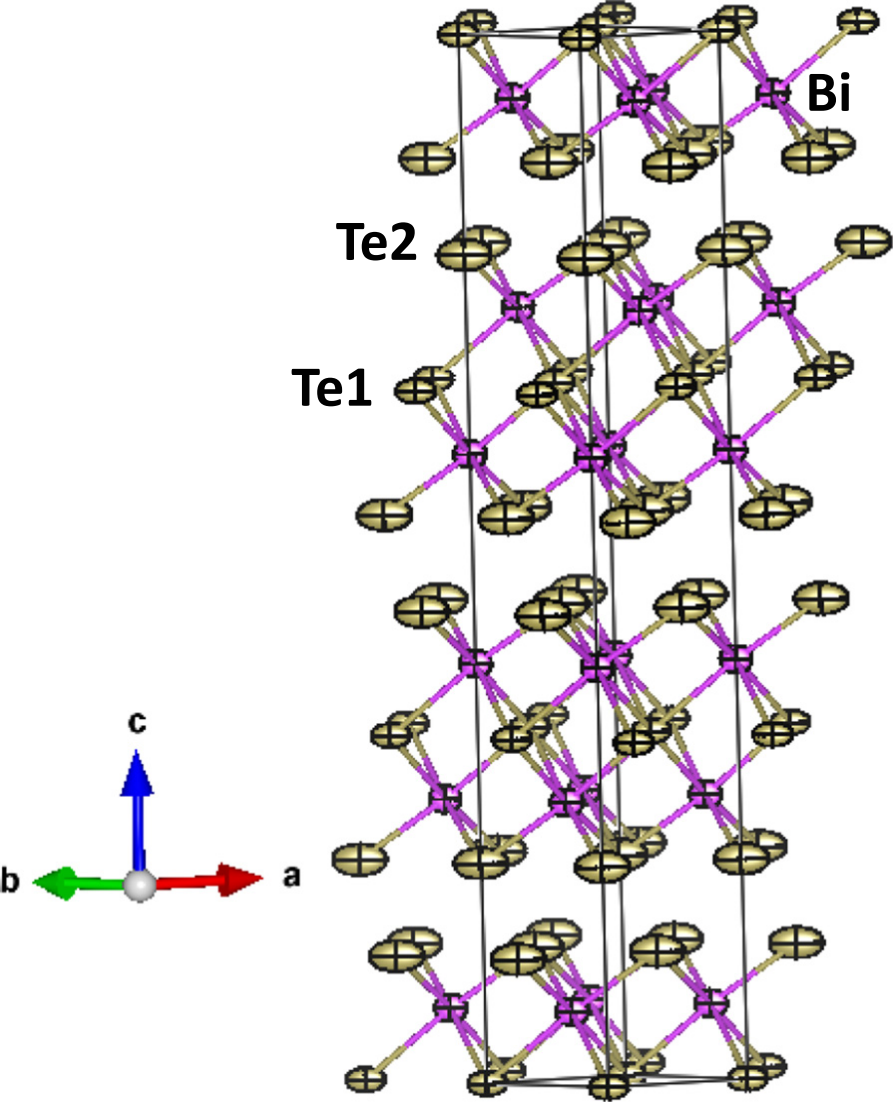
View of the layered crystal structure of Bi_2_Te_3_ with the displacement ellipsoids directed along the [1 1 0] direction, i.e., coplanar with the covalent layers

Figure 2 illustrates the layered crystal structure of this material, each layer being formed by a five-fold stacking sequence of covalently bonded Te2-Bi-Te1-Bi-Te2 atoms, whereas the interatomic forces between adjacent layers (Te2-Te2 interactions) are mainly van der Waals type. Te1 is coordinated to 6 (Bi) atoms at distances of 3.253(4) Å whereas terminal Te2 atoms are covalently bonded to only 3 (Bi) atoms at 3.061(5) Å, with the non-bonding electron pairs directed to the interlayer spacing. Bi is octahedrally coordinated to three Te1 plus three Te2. Additionally, the analysis of the neutron data yielded accurate anisotropic displacement factors for all the atoms, displayed in Fig. 2. It is remarkable that both Bi and Te atoms show flattened displacement ellipsoids with the longest semi-axis directed along the [1 1 0] direction, i.e., coplanar with the covalent layers. The thermal displacements of terminal Te2 atoms are considerably larger than those of Te1, indicating a higher flexibility of the chemical bonds. The refinement of the occupancy factors of Bi and Te excludes that Bi partially replaces Te or vice versa, confirming a precise Bi_2_Te_3_ stoichiometry within the standard deviations (±0.02).

**Fig. 3:**
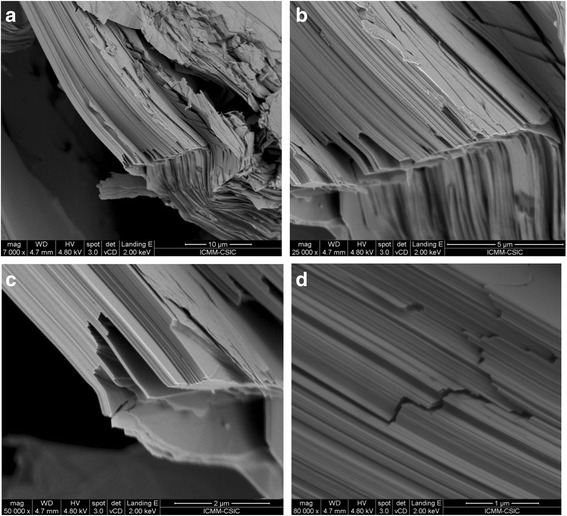
SEM images of as-grown Bi_2_Te_3_, exhibiting nanostructuration, consisting of piles of nanometric platelets (perpendicular to [0 0 1] direction). **a** ×7000, **b** ×25,000, **c** ×50,000, and **d** ×80,000 magnification, showing typical platelet thickness below 0.1 μm

Figures 3a–d illustrates the texture of the as-grown Bi_2_Te_3_ pellets collected with increasing magnification. The material consists of a stacking of nanosized sheets, each of them presumably single-crystalline, with the large surfaces perpendicular to the *c* crystallographic axis, accounting for the ease of cleavage of this material. The thickness of the individual sheets is around 0.05 μm. The thermoelectric properties of this material are strongly influenced by this nanostructuration, involving many surface boundaries that are responsible for large phonon scattering factors, bringing about low thermal conductivity, while maintaining low electrical resistivity.

**Fig. 4:**
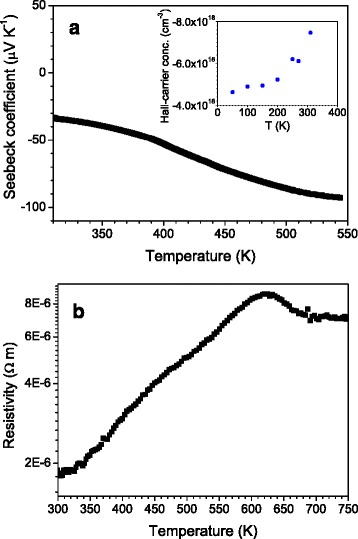
**a** Seebeck coefficient vs temperature and (**b**) thermal variation of the electrical resistivity, exhibiting a characteristic metal-like behavior in the 300–630-K temperature regime. *Inset*: hall-carrier concentration, indicating n-type carriers

The Seebeck coefficient vs temperature plot is displayed in Fig. 4a. A monotonous increment of Seebeck coefficient (in absolute value) is observed between 300 and 550 K, reaching a maximum of −93 μV K^−1^. These results were checked in numerous samples. It is worth mentioning that pristine Bi_2_Te_3_ shows an n-type semi-metallic behavior, with reported *α* values in the range of −50 to −260 μV K^−1^ [13, 22–24]. Similar *α* coefficients are measured for chemically synthesized (sintered in Ar atmosphere) samples [17, 22]. For samples prepared by ball milling and hot pressing [25], Seebeck coefficients at 300 K close to −190 μV K^−1^ are reported.

**Fig. 5:**
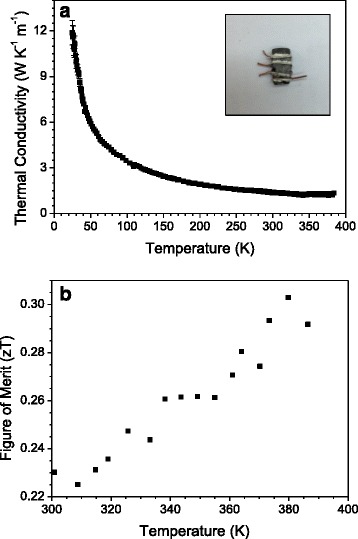
**a** Thermal conductivity vs temperature, measured by a four-probe technique. The *inset* shows one of the typical pellets, directly cut from the arc-melted ingots. **b** ZT figure of merit vs temperature

Figure 4b shows the temperature dependence of the electrical resistivity. This compound presents a semi-metallic behavior, showing the expected decrease of the conductivity when heating the sample. This behavior is consistent with the increase of the scattering of carriers as temperature increases. At 320 K, our sample presents a resistivity of 2 μΩ m, while for samples prepared by mechanical alloying and SPS or hot pressing, higher values of 30 and 7 μΩ m, respectively, have been reported [20, 25], and for chemically synthesized samples, between 13 and 5 μΩ m [22, 26]. From this point of view, our preparation conditions seem to be advantageous for the performance as a thermoelectric material. The Hall-carrier concentration (inset of Fig. 5a) increases with increasing temperature as carriers are thermally excited. The determined charge density at 310 K is 7.46°×°10^18^ cm^−3^, as expected [23]. Carrier mobility at that temperature has been assessed using *μ*_H_ = *R*_H_ * *σ*, which results in 4514 cm^2^ V^−1^ s^−1^, which is a huge mobility, consequence of the particularly low resistivity measured. This enormous electron mobility obtained may be related to the preferred orientation of the layers along the resistivity measurement direction, as Bi_2_Te_3_ is a extremely anisotropic compound, with an in-plane conductivity much higher (about three times higher) [27] than across the layers; the observed nanostructuration may account for the excellent electrical conductivity values reported here.

The thermal conductivity is presented in Fig 5a. Throughout the measurement range, *κ* decreases down to a minimum value of 1.2 W m^−1^ K^−1^ at 365 K. This is among the best (lowest) values reported in literature for the Bi_2_Te_3_ system [13], typically around 0.9 W m^−1^ K^−1^. Such a low value is an excellent compromise with the extremely high electronic conductivity, which implies a high electrical contribution to the thermal conductivity. In the present case, the relatively low thermal conductivity despite a high electrical conductivity is probably related to the nanostructured nature of the samples, which leads to a strong phonon scattering in grain (sheets) boundaries. The as-grown sample is highly textured, but it is far from an epitaxial structure or a single-crystal-like arrangement of grains. Therefore, many grain boundaries (between adjacent layers or blocks of layers) are found along the phonon paths. This is particularly effective to boost the phonon scattering at the nanoscale, and compensates for the enhanced electrical conductivity. Nanostructured systems obtained by different physicochemical procedures indeed have slightly lower thermal conductivities, in the case of ball-milling and hot-pressing procedures, of 1.2 W m^−1^ K^−1^ at 330 K [25] and for chemically prepared samples of 0.8 W m^−1^ K^−1^ at 380 K [22], but always in systems with significantly higher electrical resistivity.

Finally, the thermal evolution of the ZT figure of merit, for the above-described transport magnitudes, reaches a maximum of 0.30 at 400 K; at 500 K, it could reach 0.6, if we assume that *κ* remains unchanged in the 400–500-K range. This is a standard value normally reported in the n-type Bi_2_Te_3_ system; here we propose a straightforward, cost-effective preparation procedure, yielding polycrystalline specimens presenting a highly textured nanostructure, effectively enhancing phonon scattering and yielding a minimized thermal conductivity despite the large electrical conductivity, in robust pellets that can be directly handled and integrated into thermoelectric devices.

## Conclusions

Polycrystalline specimens of Bi_2_Te_3_ prepared by arc melting present a highly textured nanostructure, effectively enhancing phonon scattering and yielding a minimized thermal conductivity, in robust pellets that can be handled for applications. Additionally, a record-low value of the resistivity is observed, resulting from the preferred orientation of the layers along the resistivity measurement direction, as Bi_2_Te_3_ is an extremely anisotropic compound, with a better in-plane conductivity; also, it is essential for a good connection between the nanocrystalline grains. Both features are extremely attractive for useful thermoelectric materials. The crystal structure determined from neutron diffraction exhibits unreported features, in particular, large, anisotropic, displacement factors for terminal Te2 atoms, located in the boundary of the layers that compose the structure.

## Abbreviations

NPD: neutron powder diffraction
PPMS: Physical Properties Measurement System
RT: room temperature
SEM: scanning electron microscopy
XRD: X-ray diffraction
